# Somatic complaints after ICU survival: results of a post-ICU aftercare program

**DOI:** 10.1186/cc14634

**Published:** 2015-03-16

**Authors:** D Ramnarain, W Schapendonk, I Gnirrip, G Van der Nat, A Rutten, N Van der Lely

**Affiliations:** 1 St.Elisabeth Hospital Tilburg, the Netherlands

## Introduction

Critical illness today is well recognized as being associated with new or worsening physical impairment, diminished mental health and cognitive dysfunction. We studied the scope of somatic complaints in ICU survivors 4 to 6 months after ICU treatment.

## Methods

Patients who were treated in our ICU from 1 January 2013 until 31 December 2013, for 5 or more days, were invited to visit our ICU aftercare clinic. Six weeks after ICU discharge a letter of invitation together with a health-related questionnaire, the Hospital Anxiety and Depression Scale questionnaire [1] and Impact of Event Scale Revised questionnaire [2], was sent. Patients were asked to return the questionnaires before visiting our clinic. The main purpose of the post-ICU aftercare was to screen for somatic complaints, mental health and cognitive dysfunction. If necessary, further examination or treatment was advised. All data were retrospectively analyzed.

## Results

Ninety-seven patients visited our aftercare program in 2013. Median time after ICU discharge and visit to our after care clinic was 165 days. Twenty-five patients died after ICU discharge. Fifty-four patients were excluded because of various reasons; that is, language barrier, psychiatric illness, mental handicap, hospital admittance elsewhere, great distance. Seventy patients (81.4%) had somatic complaints influencing daily performance and quality of life. Fatigue (74.4%), muscle weakness (48.8%), dyspnea (34.9%), impairment of daily activity (81.4%), pain (38.4%) and weight loss (33.3%) were the most frequently reported complaints. Pain was most reported in patients with subarachnoid hemorrhage (27.3%), multitrauma (15.2%) and pneumonia (12.1%). Pain was most localized in the head (15.6%), one or both legs (15.6%), back (10.9%), shoulder (9.3%), hip (9.3%) and thorax (6.3%). Muscle weakness, fatigue, dyspnea, impairment of daily activity, pain and hoarseness were associated significantly with PTSD and HAD. There was no significant difference in somatic complaints between men and women.

## Conclusion

Somatic complaints after ICU discharge are frequently reported in our post-ICU aftercare patients, influencing daily performance and quality of life. Patient-centered research and treatment focusing on somatic complaints is of great importance.
